# Case report: Indolent drug-related pneumonitis with alectinib therapy in the treatment of non-small cell lung cancer

**DOI:** 10.3389/fphar.2022.944685

**Published:** 2022-11-01

**Authors:** Xianmeng Chen, Daqing Xia, Xuqin Jiang, Lejie Cao, Jay H. Ryu, Xiaowen Hu

**Affiliations:** ^1^ Department of Pulmonary and Critical Care Medicine, The First Affiliated Hospital of USTC, Division of Life Sciences and Medicine, University of Science and Technology of China, Hefei, Anhui, China; ^2^ Division of Pulmonary and Critical Care Medicine, Mayo Clinic, Rochester, MN, United States

**Keywords:** alectinib, anaplastic lymphoma kinase, lung cancer, drug-related pneumonitis, interstitial lung disease

## Abstract

Molecular targeting therapy is becoming the standard of care for some patients with anaplastic lymphoma kinase (ALK)-rearranged lung adenocarcinoma. Drug-related pneumonitis (DRP) has been identified as an infrequent but potentially severe adverse effect. Herein, we report a 50-year-old woman with ALK-rearranged advanced lung adenocarcinoma who developed interstitial lung disease associated with alectinib therapy. At 102-day of treatment, chest CT revealed scattered ground glass opacities (GGOs) involving both lungs. Since she was asymptomatic and alectinib provided a beneficial tumor treatment response, alectinib therapy was continued. However, 2 months later, she presented with progressive dyspnea and diffuse GGOs on chest computed tomography. There was no evidence for infection or other etiologies for her lung complication. Alectinib was discontinued and steroid therapy was initiated which was followed by improvement in respiratory symptoms and chest CT findings; DRP was diagnosed. Although rare, alectinib therapy can cause DRP of indolent onset.

## Introduction

With an estimated 2.2 million new cases and 1.8 million deaths, lung cancer is the second most commonly diagnosed cancer and the leading cause of cancer death globally in 2020 (Sung et al., 2021), of which non-small cell lung cancer (NSCLC) accounts for about 85% (Herbst et al., 2018). As a driver mutation, anaplastic lymphoma kinase (ALK)-rearrangement accounts for 2%–7% of all cases of NSCLC (Golding et al., 2018). Alectinib, a second-generation ALK inhibitor, was recommended by the National Comprehensive Cancer Network (NCCN) as first-line therapy for patients with ALK-positive NSCLC (Ettinger et al., 2018). However, drug-related pneumonitis (DRP) caused by use of ALK inhibitors has been identified as an infrequent but potentially severe adverse effect. Several cases of DRP have been reported with the first-generation ALK inhibitor, crizotinib [(Wu et al., 2018); (Yoneda et al., 2017)]. Herein, we report a case of interstitial lung disease (ILD) of indolent onset in a patient with ALK rearrangement NSCLC receiving alectinib therapy, a second-generation ALK inhibitor.

## Case presentation

**FIGURE 1:**
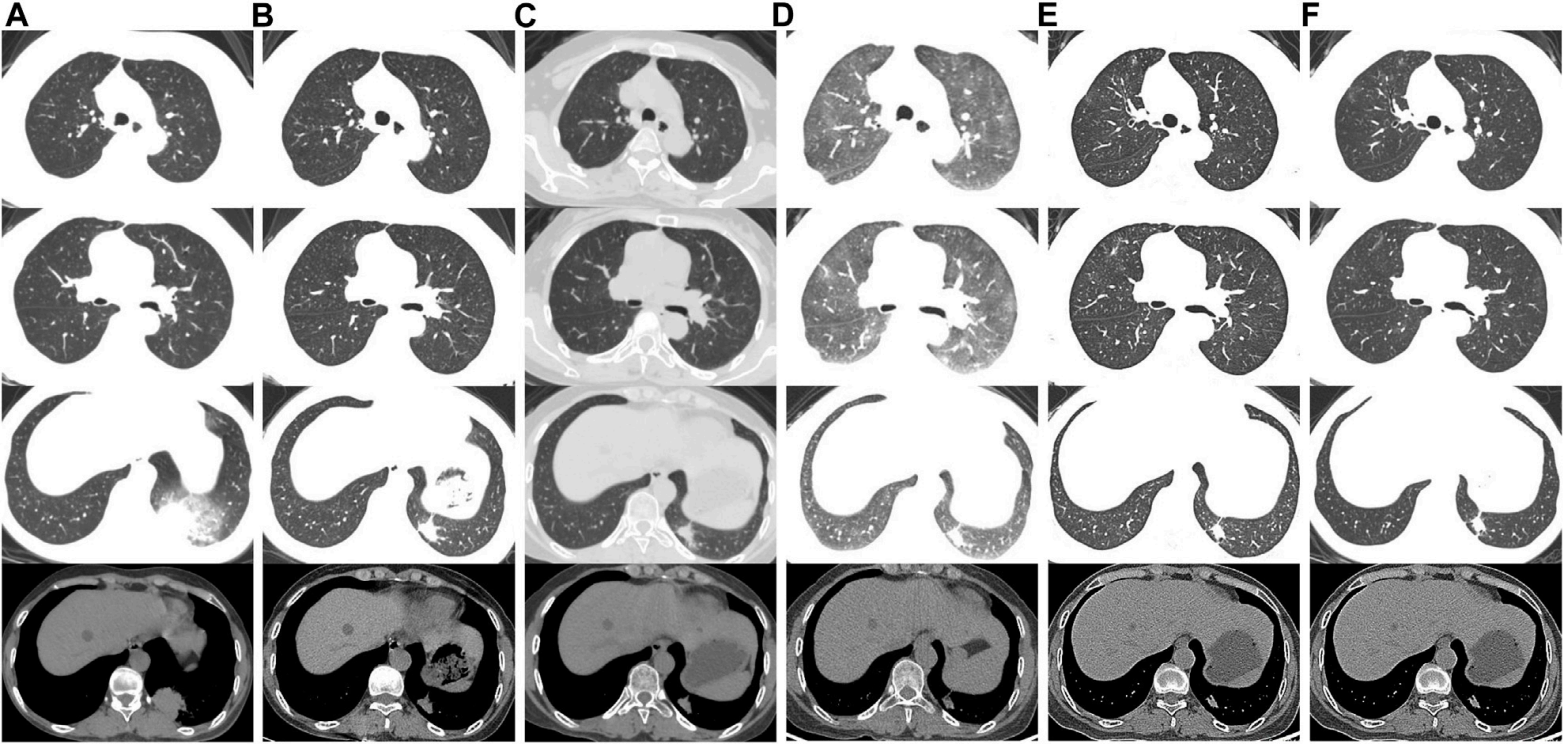
Chest CT scans of the patient**. (A)** Scans performed before alectinib therapy. **(B)** On the 30th day of alectinib therapy, chest CT showed tumor shrinkage. **(C)** On the 102nd day after alectinib therapy, chest CT showed scattered GGOs involving both lungs and tumor shrinkage. **(D)** On the 185th day of alectinib therapy, chest CT showed diffuse GGOs involving both lungs. **(E)** GGOs gradually improved after discontinuation of alectinib. **(F)** At the latest follow-up (2 months later), chest CT showed no recurrence of interstitial lung disease.

A 50-year-old female farmer, presented to the department of Rheumatology in our hospital for swelling of both lower extremities and pain in both knees and ankles for half a year. She had no relevant past medical history and was a lifelong nonsmoker with no history of alcohol intake. She denied any history of relevant environmental or occupational exposures. Autoimmune serologic screening for connective tissue diseases yielded negative results. Chest computed tomography (CT) revealed a lung mass in the left lower lobe (Figure 1A). Subsequently, CT-guided biopsy showed ALK rearrangement, lung adenocarcinoma. Metastases were found in bone and brain. She was diagnosed with stage IV (cT2N0M1c) ALK-rearranged NSCLC. Since the autoimmune serologic screening for connective tissue diseases yielded negative results, and the patient had bone metastases from lung adenocarcinoma, it was considered that the swelling of both lower extremities and pain in knees and ankles were related to pulmonary hypertrophic osteoarthropathy secondary to lung cancer. Therefore, she was started on first-line alectinib therapy at 600 mg twice daily.

One month after alectinib administration, chest CT showed tumor shrinkage (Figure 1B) and the swelling of both lower extremities and pain in both knees and ankles were significantly reduced. However, 102 days after initiation of alectinib therapy, chest CT revealed new scattered ground glass opacities (GGOs) involving both lungs (Figure 1C). Alectinib-related ILD Grade 1 (Common Terminology Criteria for Adverse Events version 5.0) was suspected (Cancer Therapy Evaluation Program, 2017). Since she was asymptomatic and alectinib provided a significant therapeutic response, we chose to continue alectinib therapy with careful monitoring.

On the 185th day of alectinib treatment, she was admitted to our department with progressive dyspnea and chest CT showed diffuse GGOs in both lungs (Figure 1D). On physical examination, the patient’s temperature was 36.8°C, pulse 103 beats per minute, respiratory rate 20 beats per minute, blood pressure 128/92mmHg, and oxygen saturation 92% on ambient air. The lungs were clear to auscultation, and inspiratory fine crackles were audible in both lower lungs. Cardiac examination revealed tachycardia, regular rhythm. Abdomen was soft and nontender. There was no lower extremity edema. An arterial blood gas analysis on supplemental oxygen *via* nasal cannula at flow rate of 2 l/min revealed a PaO_2_ of 78 mmHg, a PaCO_2_ of 43.6 mmHg, and a pH of 7.432. Laboratory findings including serum levels of C-reactive protein (<0.5 mg/l), procalcitonin (<0.10 ng/ml), white blood cell count (4.95 x10^3^/ml), neutrophil percentage (67.8%), and eosinophil percentage (5.4%) were within the normal range. Other laboratory findings, including indicators of hepatic and renal function, and autoantibodies were unremarkable. Furthermore, cardiac workup including brain natriuretic peptide level and echocardiography was normal. An extensive search for pathogens, including respiratory viruses, bacteria, and fungi, was negative on the bronchoalveolar lavage fluid (BAL) using metagenomics next-generation sequencing method. After being diagnosed with NSCLC, she had never taken any medications or supplements except for oral alectinib. Based on these results, alectinib-related ILD Grade 3 (Common Terminology Criteria for Adverse Events version 5.0) was suspected (Cancer Therapy Evaluation Program, 2017).

Alectinib was discontinued, and intravenous methylprednisolone (40 mg/day) treatment was instituted. One week later, her dyspnea was resolved with no need for supplemental oxygen. Chest CT revealed near-complete resolution of GGOs (Figure 1E). The patient was discharged at 15 days after admission. Oral prednisolone was administered at 20 mg/day, tapered, and stopped after 2 weeks. Four weeks after discontinuation of alectinib, the MRI of the brain showed new lesions in the bilateral parietal lobe and left occipital lobe. The patient declined treatment with another ALK inhibitors such as lorlatinib due to concerns over potential adverse effects and family’s financial difficulties. Therefore, following a careful discussion of the potential risks and alternatives, she started on second-line therapy with lobaplatin 50mg/m2 plus pemetrexed 500 mg/m2. At the recent follow-up (2 months later), she had no symptoms of dyspnea with no recurrence of ILD in chest CT (Figure 1F). The swelling of both lower extremities and pain in both knees and ankles were completely resolved.

## Discussion and conclusions

To our knowledge, this is the first reported case of alectinib-related ILD that manifested an indolent onset and gradual progression during continued alectinib therapy but completely resolved after discontinuation of alectinib. In most other reports, alectinib was discontinued immediately after the occurrence of ILD, and the ILD improved sequentially (Table 1) (Ikeda et al., 2015; Yamamoto et al., 2015; Nitawaki et al., 2017; Gadotti et al., 2021; Huang et al., 2021; Myall et al., 2021; Zhu et al., 2021). Two patients were reported to have continued alectinib treatment after developing asymptomatic ILD without exacerbation of ILD ((Nitawaki et al., 2017); (Hwang et al., 2019)). In our report, although this patient manifested increasing pulmonary infiltrates during 2 months of continued alectinib therapy after initial detection of possible DRP, complete resolution of DRP occurred with cessation of alectinib and glucocorticoid therapy.

**TABLE 1:** Literature review of alectinib-related interstitial lung disease.

Case No	Race/year	Demographics	Time to Onset of ILD^*^	Symptoms	Lung Abnormalities on HRCT	Treatments and Outcomes of ILD	Sequential Treatments and Outcomes of Lung Cancer
1 (Yamamoto et al., 2015)	Japanese/2015	86years/Female	215 days	Dyspnea, pyrexia	GGO, consolidations	Discontinue alectinib and corticosteroid. Improved	NA
2 (Ikeda et al., 2015)	Japanese/2015	75years/Female	102 days	NA	GGO, patchy	Discontinue alectinib. Improved	NA
3 (Nitawaki et al., 2017)	Japanese/2017	57years/Male	33 days	NA	GGO	Discontinue alectinib. Improved	Alectinib. NA
4 (Nitawaki et al., 2017)	Japanese/2017	64years/Female	12 months	Cough	GGO	Continue alectinib. Improved	Alectinib. NA
5 (Myall et al., 2021)	Asian/2018	80years/Male	18 days	Dyspnea, chest pain	GGO	Discontinue alectinib and corticosteroid. Improved	Lorlatinib. Died 7 months after tumor diagnosis
6 (Myall et al., 2021)	Caucasian/2018	66years/Female	26 days	Dyspnea	GGO	Discontinue alectinib and corticosteroid. Improved	Lorlatinib. Partial response of tumor for >10 months
7 (Hwang et al., 2019)	American/2018	46years/Female	28 days	Dyspnea, cough	Septal thickening and bilateral “crazy paving” pattern	Continue alectinib and corticosteroid. Improved	Alectinib. NA
8 (Gadotti et al., 2021)	Brazilian/2018	54years/Male	32 days	Dyspnea	Diffuse reticular interstitial opacities	Discontinue alectinib, corticosteroid and mechanical ventilation. Improved	Alectinib. NA
9 (Huang et al., 2021)	Chinese/2020	76years/Female	31 days	Dyspnea	GGO, patchy	Discontinue alectinib, corticosteroid and mechanical ventilation. Improved	Alectinib. Stable disease of tumor for 16 months
10 (Zhu et al., 2021)	Chinese/2021	65years/Male	27 days	Dyspnea	GGO	Discontinue alectinib and corticosteroid. Improved	Crizotinib. Stable disease of tumor for 6 months

^*^Time to onset is defined as the duration in days or months from initiation of alectinib therapy to presentation with interstitial lung disease; ILD, interstitial lung disease; GGO, ground glass opacity; NA, not available.

Alectinib, a second-generation ALK inhibitor, is a key drug for treating patients with ALK-positive NSCLC. A pooled analysis showed that alectinib had good efficacy against brain metastases in ALK-positive NSCLC, because it demonstrated a high brain-to-plasma ratio and was transported independently of the efflux transporter, P-glycoprotein (Gadgeel et al., 2016). Therefore, our patient was treated with alectinib as the first-line treatment due to the presence of ALK-positive and brain/bone metastases.

Use of molecular-targeting agents has increased the frequency and broadened the spectrum of lung toxicity, particularly in patients with cancer. Lung toxicity has been reported in patients taking ALK inhibitors such as crizotinib, ceritinib, lorlatinib and alectinib, with incidence rates of 1.8%, 1.1%, 1.8% and 2.6%, respectively (Pellegrino et al., 2018). DRP (interstitial lung disease) is the most common lung toxicity. The symptoms of DRP are nonspecific and some patients may be asymptomatic even in the presence of diffuse pulmonary opacities. DRP can manifest various histologic patterns and, accordingly, diverse chest CT findings. Some of the commonly encountered patterns include interstitial pneumonia either as acute interstitial pneumonia (diffuse alveolar damage) or transient lung abnormality (simple pulmonary eosinophilia), subacute interstitial disease (organizing pneumonia and hypersensitivity pneumonitis), and chronic interstitial disease (nonspecific interstitial pneumonia) (Johkoh et al., 2021). It is difficult to differentiate pulmonary infiltrates related to DRP from other causes such as infections, pulmonary hemorrhage, pulmonary edema, radiation-induced pneumonitis, and metastases. In general, DRP is suspected when the following criteria are met: 1) exposure to the causative drug; 2) development of pulmonary infiltrates; 3) meticulous exclusion of all other possible causes; 4) cessation of exposure resulting in measurable improvement of symptoms and imaging abnormalities; and 5) rechallenge (often not advisable) causing worsening (Skeoch et al., 2018). Our patient meted criteria 1 to 4, and Yamamoto et al, (2015) reported a patient with alectinib-induced ILD who had GGO and the symptom of progressive dyspnea, similar to our patient.

Currently, the precise mechanisms and risk factors associated with alectinib-related ILD are not fully understood. Some risk factors for DRP associated with other molecularly targeted drugs have been identified. Gemma et al. showed that concurrent/previous ILD, emphysema or chronic obstructive pulmonary disease, lung infection, smoking history, and shorter interval from initial cancer diagnosis to the start of treatment (<360 days) to be significant risk factors for developing ILD in patients receiving erlotinib therapy (Gemma et al., 2014). A study from Japan demonstrated that age 55 years or older, Eastern Cooperative Oncology Group performance status 2-4, smoking history, previous or concomitant ILD, and comorbid pleural effusion to be statistically significant risk factors for crizotinib-related pneumonitis (Gemma et al., 2019). Whether molecular targeting drugs-related DRP has a similar pathophysiology remains to be explored.

At present, the optimal management of alectinib-related ILD remains unclear. The drug labeling of alectinib recommends immediate discontinuation of the drug in cases of suspected DRP, and permanent discontinuation of the drug if no other underlying causes of ILD or non-infectious pneumonia are identified. However, in previous reports, patients did not redevelop DRP after restarting or reducing the dose of alectinib ((Nitawaki et al., 2017); (Gadotti et al., 2021); (Huang et al., 2021); (Hwang et al., 2019)). Furthermore, there were several case reports describing the successful use of crizotinib or lorlatinib in patients with ALK-rearranged NSCLC who have recovered from DRP secondary to alectinib therapy [(Myall et al., 2021); (Zhu et al., 2021)]. Therefore, further studies will be required to determine whether changing to another ALK inhibitors, restarting alectinib, or using alectinib combined with glucocorticoid therapy may be an option after the symptoms of alectinib-related ILD subside.

Both pulmonologists and oncologists should be aware of the possibility of DRP in patients receiving alectinib. Once alectinib-related pneumonitis is suspected, discontinuation of the drug and steroid treatments should be considered.

## Data Availability

The original contributions presented in the study are included in the article/supplementary materials, further inquiries can be directed to the corresponding author.
